# Editorial: Digital Linguistic Biomarkers: Beyond Paper and Pencil Tests

**DOI:** 10.3389/fpsyg.2021.752238

**Published:** 2021-09-16

**Authors:** Gloria Gagliardi, Dimitrios Kokkinakis, Jon Andoni Duñabeitia

**Affiliations:** ^1^Department of Classical Philology and Italian Studies, University of Bologna, Bologna, Italy; ^2^Department of Swedish, Faculty of Humanities, University of Gothenburg, Gothenburg, Sweden; ^3^AgeCap, The Centre for Ageing and Health, Gothenburg, Sweden; ^4^Centro de Investigación Nebrija en Cognición, Nebrija University, Madrid, Spain; ^5^UiT The Arctic University of Norway, Tromsø, Norway

**Keywords:** linguistic-based diagnosis, natural language processing, clinical linguistics, computational linguistics, speech processing and recognition, machine learning, computer-aided diagnosis, linguistic biomarkers

Over the last decades, a growing body of linguistic studies have been devoted to the clinical domain (Perkins, 2011), while the amount of experimental linguistic research focusing on neuroscience and mental health has increased exponentially during the last few years.

Considering that many of the factors underlying cognitive and neuropsychiatric disorders may yield to late symptoms that are hard to foresee, it is often difficult to predict the existence of a presence or risk of a disease, as well as the disease's trajectory. In this context, interdisciplinary approaches gain increasing popularity, and the analysis of complex behavior—such as speech and language—emerges as a natural candidate to identify and analyse the extent to which a given neuropathology can impact the cognitive system at the very early stages. In this context, the development of cognitive evaluation and intervention tools focusing on linguistic biomarkers becomes a critical scientific arena both in and outside the clinic and laboratory (see Petrizzo and Popolo, 2020).

Recent international research has demonstrated that automated collected and analyzed quantitative linguistic features, easily extractable from a patient's verbal productions, can be very useful in separating people with various cognitive or mental impairment from healthy subjects, even at a very early stage (see Bedi et al., 2015), and even to predict the outcomes of clinical interventions (see Carrillo et al., 2018). In this line, machine learning-based language technology methods and tools based on artificial intelligence are particularly promising to address this task (Locke et al., 2021; Sigman et al., 2021). Indeed, subtle language disruptions can be employed as *digital linguistic biomarkers*, namely objective, quantifiable behavioral data that can be collected and measured by means of digital devices, allowing for a low-cost pathology detection, classification and monitoring. Compared to classical pen-and-paper neuropsychological tests, the use of these instruments shows many advantages—such as its non-intrusive and time-effective application—providing not only offline, but also online measures that serve as a proxy for cognitive processing and its underlying mechanisms.

The aim of the Research Topic *Digital Linguistic Biomarkers: Beyond Paper and Pencil Tests* is to provide a state-of-the-art overview of this multidisciplinary and constantly evolving area of research, bringing together contributions from different quarters of the cognitive sciences. The collection comprises one systematic review, six original research papers, and one opinion paper. The articles are based on empirical and theoretical research from several disciplines (i.e., linguistics, psychology, Artificial Intelligence), and they tackle a range of developmental and acquired disorders. Most probably, dementia assessment has been one of the most rapidly evolving domain of Natural Language Processing (NLP) application for medical science (Petti et al., 2020), but this approach is spreading rapidly through the community, with encouraging results on both developmental and acquired pathologies, as shown in the current article collection (i.e., autism, developmental language disorder, attention-deficit hyperactivity disorder, Alzheimer's disease and mild cognitive impairment, or Parkinson's disease). Furthermore, this Research Topic covers a variety of test languages showing the degree of internationalization of the research on the analysis verbal productions (i.e., English, Italian, German, and Japanese).

In what follows we briefly describe the 8 articles, to help the reader navigate the volume. Martínez-Nicolás et al. open this special issue providing a systematic review of automatic voice and speech analysis of patients with Alzheimer's Disease (AD) and Mild Cognitive Impairment (MCI). These computational techniques have gained increasing popularity over the last 10 years as cost-effective and reliable methods for detecting dementia. The authors critically evaluate the quality of the evidence on this subject, to determine what linguistic features characterize these clinical conditions, which is the most effective task for eliciting oral language, and the overall diagnostic accuracy of this approach. In a similar vein, the original research article by Yamada et al. investigates through a tablet-based application whether speech responses to daily life questions could be used to differentiate elderly patients with MCI from cognitively healthy controls, and compare this approach with others based on conventional neuropsychological tasks. The authors argued that despite daily life questions may elicit weaker—but statistically discernible—differences than neuropsychological settings, combining them could help develop reliable, less burdensome health-monitoring technologies for early detection of AD.

Two of the articles of the volume deal with Parkinson's Disease (PD). Maffia et al. address the validity of %V (i.e., vowel percentage) and VtoV (i.e., the mean interval between two consecutive vowel onset points) for the identification of rhythm variation in early-stage PD speech. Their results confirmed %V as a useful cue for early-stage PD speech characterization. Moreover, the study demonstrates that reading tasks are more effective than spontaneous speech for the detection of these rhythmic variations. The experimental study by Jain et al. follows up on this and applies deep learning-based speech processing to differentiate voice features of PD patients before and after dopaminergic medication. Both proposed methods—personalized Convolutional Recurrent Neural Networks (p-CRNN) and Phone Attribute Codebooks (PAC)—show good accuracy in detecting voice qualities that are amenable to treatment. Thus, these techniques may guide the personalized evaluation of the overall motor state and the monitoring of therapy response.

Next, the contribution by Cho et al. presents the implementation of an automated language processing pipeline in a standardized neuropsychological task (i.e., the letter-guided fluency task).

The paper illustrates how the proposed approach can be used to characterize the acoustic, lexical, and semantic features of words produced by healthy young speakers. This rich set of language characteristics—which cannot be extracted manually without massive effort—strongly enhances the informativeness of the conventional paper-and-pencil test. The authors propose the possible extension of this method to the analysis of the verbal productions of neurodegenerative patients.

Moving to developmental disorders, Gale et al. present a tablet-based child language assessment tool and the dataset collected through this instrument. Their assessment framework explores four expressive language tasks (i.e., expressive vocabulary, word structure, recalling sentences, and formulated sentences), and relies on a deep neural network (DNN)-based model for the estimation of the scores directly from the transcripts. The study supports the feasibility of computerized approaches to help clinicians tasked with diagnosing speech and language impairments in children. In a related vein, Adams et al. developed a measure of lexico-semantic similarity which could be applied to children's conversational language without requiring a reference transcript. The findings of the work indicate that NLP methods can be effectively used to identify semantic coherence weaknesses that characterize children with Autism Spectrum Disorder.

Finally, the opinion paper by Tapia and Duñabeitia discusses the relationship between personalized cognitive stimulation and the improvement of language skills taking into consideration the impact of new technologies. The paper makes an important contribution concerning the role of digital linguistic biomarkers for the development of data-informed ecologically valid adaptive cognitive stimulation programs and platforms.

In conclusion, we hope the present Research Topic will help to shed light on these new research perspectives. We also believe that these novel techniques and applications may be of great value for clinicians and practitioners.

## Author Contributions

All authors listed have made a substantial, direct and intellectual contribution to the work, and approved it for publication.

## Funding

This research has been partially funded by grants PGC2018-097145-B-I00 and RED2018-102615-T from the Spanish Government; H2019/HUM-5705 from the Comunidad de Madrid; AgeCap, The Centre for Ageing and Health, Gothenburg, Sweden.

## Conflict of Interest

The authors declare that the research was conducted in the absence of any commercial or financial relationships that could be construed as a potential conflict of interest.

## Publisher's Note

All claims expressed in this article are solely those of the authors and do not necessarily represent those of their affiliated organizations, or those of the publisher, the editors and the reviewers. Any product that may be evaluated in this article, or claim that may be made by its manufacturer, is not guaranteed or endorsed by the publisher.
